# Impact of Summer Cattle Grazing on the Sierra Nevada Watershed: Aquatic Algae and Bacteria

**DOI:** 10.1155/2012/760108

**Published:** 2012-02-21

**Authors:** Robert W. Derlet, John R. Richards, Lidia L. Tanaka, Curtis Hayden, K. Ali Ger, Charles R. Goldman

**Affiliations:** ^1^Department of Environmental Science and Policy and the John Muir Institute of the Environment, University of California, Davis, CA 95616, USA; ^2^Department of Emergency Medicine, University of California Davis Medical Center, 4150 V Street, PSSB 2100, Sacramento, CA 95817, USA

## Abstract

*Introduction*. We evaluated periphytic algal and microbial communities to assess the influence of human and cattle impact on Sierra water quality. *Methods*. 64 sites (lakes and streams from Lake Tahoe to Sequoia National Park, California) were sampled for suspended indicator bacteria and algae following standardized procedures. The potential for nonpoint pollution was divided into three categories: cattle-grazing areas (*C*), recreation use areas (*R*), or remote wildlife areas (*W*). *Results*. Periphyton was found at 100% of *C* sites, 89% of *R* sites, but only 25% of *W* sites. Eleven species of periphytic algae were identified, including *Zygnema*, *Ulothrix*, *Chlorella*, *Spirogyra*, mixed Diatoms, and *Cladophoria*. Mean benthic algae coverage was 66% at *C* sites compared to 2% at *W* sites (*P* < 0.05). The prevalence of *E. coli* associated with periphyton was 100% at *C* sites, 25% of *R* sites, and 0% of *W* sites. Mean *E. coli* CFU/gm of algae detected was: *C* = 173,000, *R* = 700, *W* = 0. (*P* < 0.05). Analysis of neighboring water for *E. coli* bacteria >100 CFU/100 mL: *C* = 91%, *R* = 8%, *W* = 0 (*P* < 0.05). *Conclusion*. Higher periphytic algal biomass and uniform presence of periphyton-attached *E. coli* corresponded to watersheds exposed to summer cattle grazing. These differences suggest cattle grazing compromises water quality.

## 1. Introduction

The Sierra Nevada mountain range (Sierra) serves as the most important watershed in California and provides 50% of surface drinking water to its people [1]. The Sierra watershed spans the eastern section of California for 600 km along a northwest-southeast axis from Mt. Lassen in the north to Tehachapi Pass in the south [2]. The total area encompasses approximately 80,000 square km, nearly the size of the state of Maine, and within this area exists a high elevation watershed occupying 26,000 square km between 1,600 m and 4,500 m elevation. As much as 200 cm of precipitation occurs annually, usually in the form of winter snow [1]. The watershed consists of large sections of conifer forests and grass meadows having scant topsoil and minimal buffering capacity for pollutants, interspersed with exposed granite or metamorphic rock [2]. The high-elevation areas are the most sensitive and delicate portions of the watershed that provides the majority of runoff into a series of foothill reservoirs.

Summer cattle grazing has occurred in high elevations of the Sierra Nevada Mountains since the late 1800s. Because of the negative impact grazing had on the water quality, it was outlawed in 1891, only to be reintroduced in 1905 after intensive lobbying by the cattle industry [3, 4]. Currently nearly 40,000 head of cattle are trucked into the high elevations of the Sierra each year for summer grazing, where they have access to thousands of small streams and lakes in sensitive high-elevation meadow areas. Areas above 1,600 m elevation are fragile and sensitive to the impact of human activities. For this reason much of this land has been set aside by the Federal Government as designated Wilderness Areas, which remain roadless, and restricts overnight use by humans. Remarkably, summer cattle grazing is permitted in these areas.

Periphyton is a mixture of an algal community that attaches to submerged surfaces in most shallow water aquatic systems, but it also includes associated bacteria and detritus. The substrate for attachment varies and can include rocks, downed tree branches, sand, and mud. Similar to suspended algae, its occurrence, biomass, and composition are dependent on the availability of nutrients, particularly dissolved nitrogen and phosphorus, and thus are a common indicator for water quality in various habitats ranging from wetlands to rivers and streams. Over the past 10 years, our research group and others have observed clusters of thick periphyton mats in specific areas of the Sierra where summer cattle grazing occurred [5, 6]. These observations are consistent with nonpoint source pollution from cattle grazing which deposits growth stimulating substances from manure. Substances such as phosphorus and nitrogen have a significant impact on aquatic life and microbial communities [7]. Although the State of California has water quality standards for suspended indicator bacteria, no standard or monitoring exists with respect to bacteria, which attach to periphyton [8]. This is concerning because microorganisms are known to attach to fresh water algae individually or as biofilms [9]. The need to study the relationship of bacteria with periphyton is further strengthened because of recent reports of *E. coli* having been described as attached to green filamentous algae in the Great Lakes area of the USA [10]. *E. coli* easily detaches from the algae, into surrounding water [10].

The growing population of California has placed high demands on the limited supply of potable water and shifted the greatest economic value of the Sierra to this end [11]. Despite this dependence on Sierra water for drinking and irrigation, little has been published on periphyton communities in high-elevation watersheds, other than Lake Tahoe [12]. The study of the biomass, taxonomy, and association of periphyton-attached bacteria in the Sierra watershed thus might provide important data, which can be used in management decisions.

Therefore, we have undertaken this study of small lakes and streams of the Sierra with the following aims: (1) to estimate the biomass of periphytic algae, (2) to determine their taxonomy, (3) to measure the quantity of heterotrophic and indicator bacteria attached to the periphyton, (4) to measure the presence of suspended aquatic bacteria neighboring the periphyton, and finally (5) to compare the above metrics between cattle grazing and noncattle areas.

## 2. Methods

### 2.1. Field Sampling Site Selection

**Figure 1:**
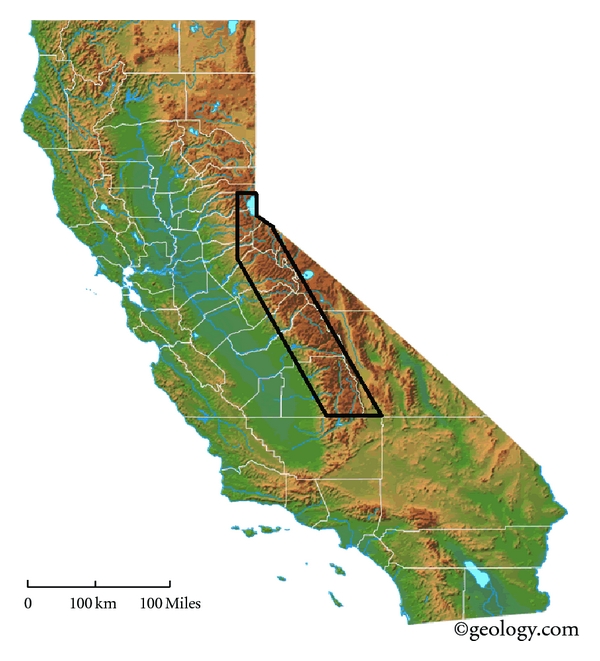


Sampling occurred along a 350 km section of the Sierra Nevada Mountains of California, between the Lake Tahoe area in the north to Sequoia National Park in the south (Figure 1). Most of this area is roadless, and all small lake and stream sampling sites were accessed by foot at distances ranging from one to 38 km from a trailhead. These sites had been analyzed in past years for heterotrophic and fecal indicator bacteria, but not for periphyton [13]. Collection sites ranged from 1,650 to 3,800 m elevation. The potential for nonpoint pollution was divided into three categories reflecting impact of the environment by absence or presence of human activities: Wildlife areas which had little or no human or domesticated animal traffic (*W*), established recreational areas with moderate-to-heavy human recreational and variable pack animal traffic (*R*), and summer cattle grazing tracts (*C*), where cattle are trucked to High Sierra meadows for 90–100 days during the summer months. Watershed impact categories were confirmed by the National Park Service representatives, USDA National Forest Service, the Central Sierra Environmental Resources Center, and other organizations.

### 2.2. Field Collections and Site Analysis

#### 2.2.1. Periphyton

Sites were analyzed from June through October 2010. The extent of underwater periphyton invasion was roughly estimated using a modified California Fish and Game Department method [14]. A one *x* one meter square was created from four attached 1 m pieces of wood carried into the field. This square was placed over the collection site, and the amount of area occupied by algae versus uncovered rock sand or mud was estimated as a percentage. A photograph was taken and later used to confirm the field estimate. Periphyton was collected by scraping it from attachment to underwater rocks, sand, or benthic mud using sterile metal forceps. We harvested the periphyton from a depth of 10 to 30 cm below the water surface to collect a sample ranging between 100 to 300 mg wet weight. Periphyton was then placed in sterile 8 mL plastic test tubes to which native water was added to 5 mL followed by two drops of Lugol's solution. A second sample of periphyton was placed in leak-proof plastic test tubes (Sarstedt, Germany) containing 5 mL of Cary-Blair transport media. If no periphyton was visible at the sampling site, a field inspection was made to include up to 0.5 km of lake or stream shore. If still no periphyton was found, rock scrapings were taken for microscopic examination, preserved with two drops of Lugol's solution in 5 mL of sample water.

#### 2.2.2. Suspended Aquatic Bacteria

Water surrounding the periphyton was collected in both Millipore total coliform and heterotrophic bacteria count samplers (Millipore Corporation, Bedford, MA). All samples were shielded from light by wrapping in aluminum foil and transported to the University's Limnology Laboratory within 72 hours of collection. All samples were collected in duplicate. To prevent deterioration from high temperatures during transport from trailhead to laboratory, samples were kept in a cooler at 5°C. This technique was repeated 5 m on both sides of the collection site and the mean of all measurements recorded.

#### 2.2.3. Controls

Both negative and positive controls were taken into the field. The negative control consisted of a collection test tube with 5 mL of Cary-Blair transport media. It was opened once during field collections to simulate sampling conditions. The positive control consisted of a tube containing 5 mL of Cary-Blair transport media inoculated prior to departure with 50,000 colony forming units (CFUs) of *E. coli*. This tube was sealed and not opened in the field. Water temperature was measured at each site using a stream thermometer (Cortland Line Company, Cortland, NY). Location and elevation were determined using US Geographical Society topographical maps, guide books, and backcountry rangers.

### 2.3. Laboratory Analysis of Water Samples

#### 2.3.1. Periphyton

Preserved periphyton was removed from the tubes and placed on microscope slides. An expert in algae taxonomy examined the samples under a microscope to identify the species of algae. A second investigator confirmed identification. The samples containing the periphyton preserved in Cary-Blair transport media were analyzed for bacteria within 12 hours of arrival. Each sample was vortexed for 5 minutes. Multiple 10 *μ*L aliquots of the solution were then plated onto four 100 mm diameter agar plates: two MacConkey (MAC) agar plates and two sheep blood agar (SBA) plates. One MAC plate and one SBA plate were incubated at 35°C and the other set at 44°C, for 24 hours. Colonies of visible bacteria on the plates were then counted and recorded as CFU. Colonies with color change from the purple indicator dye on the MAC plates were presumed Coliform bacteria from the 35°C plates; colonies incubated at 44°C were presumed to be *E. coli*. Additional standardized lab analysis of the presumed *E. coli* was performed to confirm this identification. Algae was then centrifuged, collected, and weighed. The algae weight in combination with the CFU/10 *μ*L solution was used to calculate the number of bacteria per gram of algae using the following formula: CFU/10 *μ*L × 500 divided by weight of algae in grams.

#### 2.3.2. Suspended Aquatic Bacteria

Analysis for suspended bacteria has been previously described in detail [13]. The analysis for Coliform and total bacterial counts required incubating Millipore counting plate paddles at 35°C for 48 hours. Bacterial colonies were counted then harvested and subplated for further analysis following standardized procedures. Colonies are plated onto SBA, MAC, and Sorbitol agars (Reel Inc, Lenexa, KS). Lactose-fermenting colonies from MAC plates were presumed to be Coliform bacteria and were subject to further testing. Further screening and initial identification were done by sub-plating onto eosin methylene blue (EMB Levine), cefsulodin irgasan novobiocin (CIN), and Hektoen agars. The color and morphology of the colonies were recorded. Each sample device measured bacteria for one mL of sample. This was multiplied by 100, as per standardized procedure of reporting CFU/100 mL for the heterotrophic bacteria. Coliform and *E. coli* were not quantified, and instead results reported positive or negative. To be positive the quantity would need to exceed 100 CFU/100 mL.

### 2.4. Results

**Figure 2:**
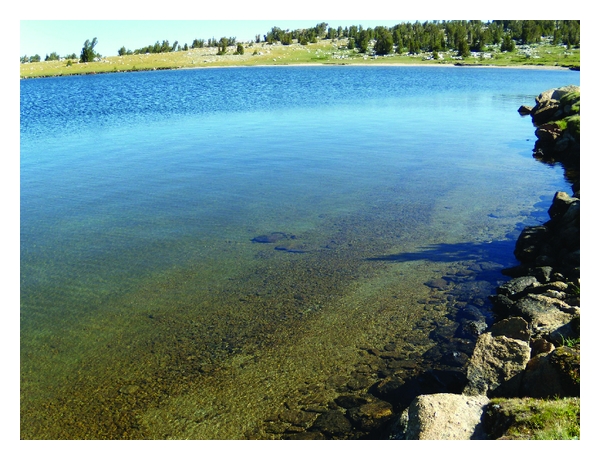


**Figure 3:**
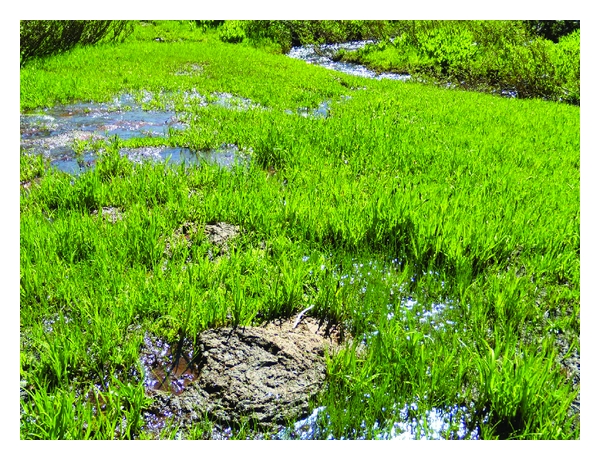


A total of 64 sites were analyzed and listed in Table 1. Periphyton was visualized in the field and collected at 48 of these sites.  Figure 2 shows a photograph of a typical *W* site, and Figure 3, a *C* site. At 16 sites no periphyton was visible to the unaided eye. Microscopic analysis of rock scrapings from these sites did not contain any recognizable periphytic algae. Twelve of these sites were from the *W* category. Field observations confirmed the oligotrophic nature of these sites, with outstanding water clarity noted. The four additional sites without periphyton were found at *R* sites. All four of these recreational sites received minimal human usage and retrospectively should have been categorized as *W*.

### 2.5. Taxonomy

Of the 48 sites where visible periphyton was collected, 12 were from *C* sites, 32 from *R* sites, and 4 from *W* sites.  Table 2 displays the species of algae identified and includes *Zygnema, Ulothrix, Chlorella, Spirogyra,* mixed Diatoms, and *Cladophora. *Only* Zygnema, Spirogyra, and *diatoms were identified at *W* sites. Only *Zygnema* and *Ulothrix* were identified in *C* areas. Benthic coverage of periphyton and estimation of biomass ranged from 0 to 90%, with highest coverage in *C* areas compared to other areas. Mean benthic coverage by use category was 66% for *C* areas, 41% for *R*, and 2% for *W *(*P* < 0.05).

### 2.6. Microbes Attached to Periphyton

All samples of periphytic algae grew out heterotrophic bacteria when plated. The mean bacterial CFU/gm of bacteria attached to periphytic algae were recorded as follows (CFU/gm): *C* = 2,014,000, *R* = 1,968,000, and *W* = 335,000 (*P* < 0.05).

 The prevalence of Coliform bacteria associated with periphyton was 100% at *C* sites, 53% at *R* sites, and 12% at *W* sites. Mean Coliform bacteria counts per gram algae were *C* = 198,000, *R* = 150,000, *W* = 39,000 (*P* < 0.05).

The prevalence of *E. coli* associated with periphyton was 100% at *C* sites, 25% of *R* sites, and 0% of *W* sites. Colony counts/gram algae were *C* = 173,000, *R* = 700, *W* = 0 (*P* < 0.05).

### 2.7. Aquatic Suspended Bacteria

Analysis of neighboring water for indicator bacteria >100 CFU/100 mL revealed the following: suspended aquatic coliforms, *C* = 100%, *R* = 25%, *W* = 0; suspended aquatic *E. coli*, *C* = 91%, *R* = 8%, *W* = 0 (*P* < 0.05).

### 2.8. Controls and Multivariant Analysis

Negative controls of Cary-Blair media taken into the field had no growth. Positive controls had a 90% survival rate of bacteria. Multivariate analysis of data found no significant differences as a result of water temperature, latitude, elevation, or distance from the trailhead.

## 3. Discussion

Little data has been published on the taxonomy of periphytic algae in the High Sierra. This study identified 11 species of periphytic algae found in high-elevation areas of the Sierra. The species are consistent with those species found in other high-elevation mountain areas of the world. In our study, periphytic algae were found at all *C* sites, but at only 4 of 16 *W* sites. This is consistent with the oligotrophic appearance and nature of these sites with absence of any obvious source of point or nonpoint pollution. All 11 algal species were found within *R* areas. Compared with *W* areas, the higher biomass of periphyton in *C* areas is not surprising, as cattle grazing contains large amounts of nitrogen, phosphorus, and other nutrients that promote algal growth [15]. Cattle allotments in the High Sierra are not fenced off from lakes and streams, so deposition of manure may occur directly into the aquatic environment or gain entry from summer thunderstorm wash off. Excess algae growth, in addition to supporting bacterial survival, has detrimental effects on the ecology of sensitive watersheds [16]. The biomass found at *R* sites, while not as high as cattle areas, was greater than *W* sites. This is may reflect nonpoint pollution in recreational areas. Although backpackers are instructed not to pollute water sources, violation of this regulation may occur. Recreational-associated algae could result from nonpoint pollution by horses and pack animals, which may defecate in or near water sources. This was the conclusion of a recent study conducted near the John Muir Trail in California which compared areas used by horses and mules versus those areas used exclusively by backpackers on foot [17]. Our field staff did notice that algae biomass was greater in those R areas with high traffic of pack animals, compared with areas only used by humans on foot.

Our findings of high numbers of heterotrophic and indicator bacteria attached to periphytic algae in Sierra recreational and cattle areas are similar to studies from another area of the United States. In the Great Lakes region, several studies found heterotrophic bacteria, fecal Coliforms, and *E. coli* attached to the green algae *Cladophora* [9, 10, 18, 19]. *Cladophora* provides protection and nutrients, which allow enteric bacteria such as *E. coli, Enterococci, Shigella, Campylobacter, *and* Salmonella *to persist and potentially flourish in the presence of the algae. Authors of these studies warn of potential public health dangers. In South Asia, several *Vibrio* species, including Cholera, attach to algae in order to survive [20]. It is highly likely that the species of green algae identified in this and prior studies support bacterial growth in a similar way [19]. The colony counts of total bacteria per gram of periphytic algae found in our study are similar to those seen in one of the Great Lakes studies [21]. Disturbance of this algae may result in release of bacteria in to the water [9]. Although most downstream water for domestic use is filtered and chlorinated, excessive bacteria loads can strain treatment systems. Furthermore, when periphytic algae detaches from its foothold, it may flow downstream clogging filtration systems, increasing the cost of such systems. A high prevalence of *Giardia* can be found in cattle manure, but any dependent association to algae in watersheds has not yet been described [22, 23].

The bacteria found in the aquatic environment surrounding the periphyton were the same found attached to the periphyton. However the prevalence of indicator bacteria was less. For example, at *R* sites, coliform bacteria was found on 53% of periphyton, but in only 25% of the water samples. This may reflect a capture effect of the bacteria onto the periphyton. The finding of a higher prevalence of indicator bacteria greater than 100 CFU/100 mL in cattle-grazed areas is consistent with prior studies [6, 13]. Derlet et al. conducted what might be considered the definitive study showing that suspended aquatic *E. coli* increases from nearly undetectable levels before cattle arrive for grazing to as high as 550 CFU/100 mL during grazing season [13]. In the current study, coliform and *E. coli* prevalence in cattle areas was much higher compared with either *R* or *W* sites. Standards for indicator bacteria vary by water board districts in California. Even though the Sierra watershed is used in domestic municipal water systems, it is regulated as “recreational water.” Eastern Sierra water standards for fecal coliforms call for less than 20 CFU/100 mL water. Western Sierra standard is 200 CFU/100 mL. Cattle grazing violates both these standards.

In parts of the United States and in other countries, summer cattle grazing has a damaging impact in sensitive watersheds [6, 24–26]. The unique geographic features of the Sierra have resulted in challenges to maintain water quantity and quality. Melting snow must pass through a fragile ecosystem prior to runoff into lowland reservoirs. Therefore, relatively small amounts of nutrient addition or habitat disturbance leads to significant impacts on nutrient flux and the aquatic ecosystems of lakes and streams. Our study has shown that nonpoint pollution associated with grazing has an impact on periphytic algae and the bacteria which attach to the algae.

Disagreements have occurred between those in the cattle industry and environmentalist groups as to the impact of cattle on water quality. In California the USDA Forest service denies cattle grazing has a negative impact on surface water quality [27]. However, decreased water quality in alpine areas downstream cattle grazing has been detected from different parts of the world including the study area [13, 25, 26]. The economic benefit of summer grazing practice is debatable [28]. A recent study showed 59% of central California ranchers felt there was either no benefit or a neutral benefit of leasing inexpensive Forest Service allotments versus private land leases in the foothills [29]. Of concern is the far greater cost to filter and disinfect water which has been polluted with cattle manure. In addition, aquatic systems subject to agricultural runoff commonly develop harmful algal blooms and subsequent algal toxins including *Microcystis* toxins [30–32]. Removing such toxins to make water acceptable for domestic systems is difficult and expensive [33]. Preventing nonpoint pollution in fragile and sensitive high elevation meadows from alpine summer cattle grazing can be achieved by prohibiting such practice and relocating cattle to more forgiving lower elevation areas [11].

Limitations exist with this study, as is true with any alpine field work. In addition to nutrients, variation in periphyton biomass and abundance can be regulated by water flow, depth, and the presence of aquatic grazers such as snails and fish [34, 35]. Thus, part of the variation in our results may have been due to differences in such controls on periphyton, which were not measured in this study. Hence, future studies should take into account the water flow, depth, and the presence of potential periphyton grazers. The presence, composition, and abundance of pathogenic bacteria attached to periphyton are a clear indicator of water quality and not necessarily dependent on the biomass of periphyton. Therefore, despite the potential variation caused by differences in water flow and grazers, the results indicate real differences in water quality indicators attributed to periphyton among the different land use categories. Finally, the distribution of periphyton at a given habitat can be naturally patchy, which may add to variability in the results. However, our sampling method minimizes the risk of subjective sampling by focusing on previously existing sites that relied on identifying the most representative segment of benthic habitat as the target site.

## 4. Conclusion

Our results indicate that summer cattle-grazed areas in alpine watersheds have increased periphytic algal biomass, attached heterotrophic bacteria, and attached *E. coli* compared with nongrazed areas. The prevalence of suspended aquatic *E. coli* in surrounding water in cattle grazed areas is significantly higher compared to non-grazed areas. These data suggest that non point pollution from cattle grazing may be a significant cause of deteriorating water quality within these source watersheds.

## Figures and Tables

**Table 1:** 

Wild sites	Recreational sites	Cattle sites
Golden Bear Lake, KC	Charlotte Lake, KC	Cottonwood Creek, Stan
West Window Creek, KC	Bullfrog Lake, KC	Boggie (High), Stan
Marie Lake, Muir	Smith Lake, Tahoe	Boggie (Mid), Stan
Silver Pass Creek, Muir	Bubbs Creek, KC	Boggie (Low), Stan
Silver Pass Lake, Muir	Rae Lake (Mid), KC	Cow Creek, Stan
Chief Lake, Muir	Rae Lake (Lower), KC	Little Walker River, Hoover
Lake Virginia, Muir	Arrowhead-Dollar, KC	Molybdenite, Hoover
Granite Lake, Yos	Dollar Lake, KC	Buckeye, Hoover
Gaylor Lake, Yos	SF Kings River, KC	Big Meadow (High), Hoover
Conness Creek, Yos	Lake 12,500 m, JMT, KC	Big Meadow (Low), Hoover
Upper Young Lake, Yos	Kearsarge Lake, KC	Summit Lake, Tahoe
Townsley Lake, Yos	Booth Lake, Yos	Bull Creek, Carson
Lake 12,248 m, KC	Fletcher Creek, Yos	
Bago Springs, KC	Fletcher Lake, Yos	
Glen Pass Spring, KC	Vogelsang Lake, Yos	
Creek from Lake 10320 m, KC	Ireland Lake, Yos	
	Ireland Creek, Yos	
	Tuolumne River (High), Yos	
	Tuolumne River (Low), Yos	
	Young Lake, Yos	
	Side Creek/Young Lake, Yos	
	Dog Lake, Yos	
	Middle Young Lake, Yos	
	Duck Lake, Muir	
	Purple Lake, Muir	
	Fish Creek, Muir	
	Squaw Lake, Muir	
	Hilgard Creek, Muir	
	Mono Creek, Muir	
	Robinson Creek, Hoover	
	Barney Lake, Hoover	
	Fremont Lake, Hoover	
	West Walker River, Hoover	
	Long Creek, Hoover	
	Toejam Lake, Emigrant	
	Silver King Creek, Carson	

KC: Kings Canyon National Park, Yos: Yosemite National Park, Muir: John Muir Wilderness Area, Hoover: Hoover Wilderness Area, Carson: Carson Iceberg Area, Stan: Stanislaus National Forest, Emigrant: Emigrant Wilderness Area, Tahoe: Tahoe National Forest, JMT: John Muir Trail.

**Table 2:** 

Noncattle sites	Cattle sites
*Zygnema *	*Ulothrix*
*Ulothrix *	*Zygnema*
*Chlorella*	
*Oedogonium*	
*Spirogyra*	
*Diatoms,*	
*Cladophora*	
*Mougeotia*	
*Didymosphenia*	
*Chlorokybus*	
*Chlorococcum*	
